# Oral health knowledge, attitudes, and behaviors during Ramadan: a cross-sectional study among adults in Qatar

**DOI:** 10.3389/froh.2026.1773185

**Published:** 2026-06-30

**Authors:** Najat Alyafei, Abdel-Salam G. Abdel-Salam, H. R. Al Mudahka, Hamda Ali AlSaadi

**Affiliations:** 1 Primary Health Care Corporation, Doha, Qatar; 2 Qatar University, Doha, Qatar

**Keywords:** dental visits, dietary habits, fasting, knowledge, oral hygiene, Ramadan, sugar consumption

## Abstract

**Objective:**

Ramadan fasting influences daily routines, dietary patterns, and oral hygiene behaviors among Muslims. Understanding how these changes affect oral health is essential for developing culturally appropriate health promotion strategies. This study examined oral health knowledge, attitudes, behaviors, and sugar consumption among adults living in Qatar during Ramadan.

**Methods:**

A cross-sectional survey was conducted among 199 adults. Data were collected using a structured self-administered questionnaire covering oral hygiene practices, dietary habits, attitudes toward oral health, and perceptions of dental care during fasting. Descriptive statistics, chi-square tests, Kruskal–Wallis tests, McNemar tests, and logistic regression analyses were applied to assess associations and predictors of oral health behaviors.

**Results:**

Participants demonstrated generally high oral hygiene awareness, with most maintaining regular brushing before and during Ramadan. However, misconceptions persisted, as 23% believed that toothpaste use invalidates fasting. Education level was the strongest predictor of oral health knowledge, and positive attitudes significantly influenced brushing frequency. Younger and less-educated individuals reported higher sugar consumption, often linked to cultural Ramadan traditions. The findings partially supported the Knowledge–Attitude–Behavior model.

**Conclusion:**

Culturally grounded and faith-aligned oral health promotion strategies are needed to address misconceptions and reduce sugar consumption during Ramadan. Integrating community-based and digital educational approaches may help sustain healthy oral hygiene behaviors throughout and beyond the fasting month.

## 1 Introduction

Oral health is a vital component of overall well-being, influencing nutrition, systemic health, self-esteem, and quality of life. The World Health Organization (1) recognizes oral diseases as major global public health challenges due to their high prevalence and strong links with chronic conditions such as diabetes, obesity, and cardiovascular diseases. Maintaining oral hygiene depends on consistent preventive behaviors including toothbrushing, flossing, and regular dental visits; however, cultural, social, and religious contexts may influence these practices, particularly during periods such as the Islamic holy month of Ramadan (2).

Ramadan is observed by Muslims worldwide through fasting from dawn to sunset for approximately 30 days, during which eating and drinking are restricted during daylight hours. The fasting period modifies daily routines, meal timing, sleep patterns, and dietary behaviors. In Qatar, where Ramadan holds strong cultural and social significance, the two main meals Iftar and Suhoor often include energy-dense and sweet foods and beverages that may influence oral health outcomes (3).

Studies have reported reductions in oral hygiene practices during Ramadan, frequently associated with misconceptions regarding whether brushing teeth or using mouthwash invalidates fasting (4, 5). Such behavioral changes, together with increased intake of sugary foods during evening meals, may elevate risks of dental plaque accumulation, halitosis, and dental caries when preventive practices are not maintained (6).

Despite global and regional recognition of these challenges, limited research has comprehensively examined the interaction between oral health knowledge, attitudes, hygiene behaviors, and dietary practices during Ramadan in Qatar. Previous studies often addressed oral hygiene habits or educational aspects separately without integrating behavioral, dietary, and psychosocial determinants (7). A holistic framework that examines these interrelated factors is therefore essential to understand how religious and cultural contexts shape oral health behaviors during fasting.

Qatar provides a unique setting for such investigation due to its diverse population, rapid urbanization, and strong adherence to religious traditions. Modern lifestyle transitions, including increased availability of processed and sugar-rich foods, coexist with enduring fasting practices that influence family routines, social gatherings, and dietary patterns. During Ramadan, these cultural and behavioral dynamics intersect, creating distinctive conditions that may affect oral health practices among adults and children alike (6–8).

Understanding oral health behaviors during Ramadan has important implications for culturally informed public health strategies. Existing oral health promotion initiatives in Qatar are often generalized and not specifically adapted to behavioral changes occurring during fasting. Addressing misconceptions through scientifically accurate and religiously aligned health education may strengthen adherence to preventive practices while respecting spiritual observance. In addition, examining parental dietary practices and their influence on children's sugar consumption can support the development of family-centered preventive interventions (8).

This study adopts a comprehensive analytical framework incorporating correlation, regression, mediation, moderation, and structural equation modeling (SEM) to explore how knowledge, attitudes, beliefs, oral hygiene behaviors, and sugar intake interact during Ramadan (9). Investigating subgroup differences by age, gender, and education may further assist in tailoring culturally sensitive public health strategies. Beyond addressing a public health gap, the study contributes to understanding how religious beliefs interact with health behavior within Islamic contexts and may support collaboration between healthcare professionals, religious scholars, and community leaders in promoting oral health during fasting (10).

Therefore, this study investigates how cultural, behavioral, and religious determinants interact to influence oral health knowledge, hygiene practices, and sugar consumption patterns during Ramadan in Qatar, providing evidence to inform culturally responsive oral health promotion strategies.

The study aims to provide a comprehensive assessment of oral health knowledge, behaviors, attitudes, and sugar consumption patterns among individuals living in Qatar during Ramadan. Specifically, the objectives are:

Assess oral health knowledge, hygiene behaviors (including brushing, flossing, and mouthwash use), attitudes, and dental visit intentions during Ramadan.

Quantify sugar consumption and examine dietary habits at Iftar and Suhoor, focusing on commonly consumed sweet foods and beverages.

Evaluate item-level responses to identify misconceptions and educational needs for targeted interventions.

Determine relationships between oral health knowledge, attitudes, hygiene behaviors, and dietary factors using correlation, regression, mediation, and moderation analyses.

Explore subgroup differences by age, gender, and education to inform culturally sensitive public health strategies.

Test an integrated theoretical framework using structural equation modeling (SEM) to identify direct and indirect pathways influencing oral health behaviors during Ramadan.

Through these objectives, the study seeks to bridge oral health education with culturally grounded behavioral understanding and generate evidence-based recommendations for oral health promotion during Ramadan and similar fasting contexts.

### 1.1 Hypothesis testing

H1: Individuals who believe toothpaste or mouthwash breaks the fast are less likely to brush during Ramadan.
H2: Higher educational attainment is associated with better knowledge regarding oral care during fasting.
H3: Individuals consuming higher amounts of sweets during Iftar and Suhoor are less likely to regulate their children’s sugar intake.
H4: Greater understanding of religious rulings is associated with improved oral hygiene behavior.
H5: Individuals who perceive sufficient support from health authorities demonstrate more positive attitudes toward maintaining oral hygiene.

These hypotheses are grounded in behavioral theory, proposing that knowledge, attitudes, and beliefs jointly influence preventive health behaviors and allowing identification of psychosocial and cultural determinants shaping oral health practices during Ramadan.

## 2 Methods

### 2.1 Study design

This research study employed a cross-sectional quantitative survey design focused on assessing oral health knowledge, attitudes, behaviors, and sugar consumption patterns among participants living in Qatar during the period of Ramadan. The cross-sectional approach was considered effective since it permitted capturing a snapshot of fasting-related oral health practices during a specified period, which facilitated making comparisons across demographic groups and behavioral dimensions. Additionally, the design was suitable for recognizing association among major variables like education level, knowledge, and oral hygiene practices; it enables assessment of the existing theoretical model's framework (11).

The survey design utilized both descriptive and analytical components to derive the needed insights. Descriptive analysis was used to provide insights into the occurrence of certain habits, misconceptions, and attitudes. On the other hand, analytical testing determines associations, differences, and predictive connections among the study variables. The design underscored cultural sensitivity through contextualization of oral health practices within the religious practice of fasting, which acknowledges both theological and behavioral impacts on health decisions. Ethical considerations were upheld throughout the study and ensured voluntary participation and anonymity of the collected data to promote authenticity and reliability in the derived findings (12).

Lastly, the partial support for the Knowledge–Attitude–Behavior (KAB) model will be explored in the current study to replicate global research trends. The logistic regression results will be utilized to certify that knowledge alone cannot be used to directly predict brushing behavior, but attitudes must be considered (with an odds ratio). This is supported by previous research (13, 14), which point out that although awareness is necessary, behavioral change requires positive reinforcement and belief validation. Likewise, Ramadan focused oral health studies have underscored that health interventions target both cognitive and affective domains for effectiveness to be realized (3). Hence, it is expected that the findings ought to affirm that health education should go beyond factual instruction to address cultural, emotional, and spiritual incentives that dictate the fasting behavior of individuals.

### 2.2 Study participants

The target participants of this study comprised adult men and women who were residing in Qatar during the Ramadan period. The sample incorporated a diversified range of nationalities, ages, occupations, and education levels to illustrate the multicultural population of Qatar. Participation was limited to respondents who were primarily Muslims and observing the Ramadan fasting ritual; they were recruited from Qatari and expatriate communities in the country. The inclusion of participants from numerous employment sectors, for instance governmental, private, and self-employed entities, augmented the dataset since it provided a wide range of socioeconomic and occupational perspectives.

Most of the target respondents were middle-aged adults since it is a demographic group that has a high likelihood of balancing professional responsibilities with family and religious commitments. This makes them relevant to studies of health behaviors during the Ramadan period. The insights derived were considered essential in providing valuable information on household-level decisions like the regulation of children's sugar intake and informing on the perceptions of oral hygiene tolerability during fasting. Hence, participant recruitment for this study was guided by accessibility and diversity principles to guarantee the capture of diversified social and cultural segments for proper representation of Qatar's population.

### 2.3 Inclusion and exclusion criteria

Participants qualified to be included in this study if they self-reported to be aged 18 years or older, were residing in Qatar at the moment, and had been observing fasting during the Ramadan period. Eligibility mandated the ability to understand and respond to an online questionnaire that was provided in either English or Arabic, which warranted accessibility for a wider range of participants. Also, those with partial or incomplete survey questionnaires were excluded to preserve analytical integrity and ensure the data collected was consistent across the participants. It is important to note that individuals aged below 18 years and those not participating in the fast for medical or personal reasons, or those reluctant to provide informed consent, were excluded from involvement in the study. Moreover, respondents with inconsistent responses or multiple skipped items were removed from the dataset during the data cleaning process. This approach was necessary to improve the reliability of statistical results by keeping only responses that were valid, complete, and coherent. Hence, the final sample size considered in this research study was 199 participants, as those who met the minimum recommended threshold, which provided adequate statistical power that help in detecting significant relationships among the variables.

### 2.4 Sampling

A convenience sampling method was utilized among voluntary respondents to collect data efficiently within the Ramadan fasting month (15). A survey link was created and distributed via digital platforms to potential participants in institutional mailing lists, community groups, and professional networks. This allowed for a wide outreach among the Qatari population. The approach was preferred for its practicality, cultural appropriateness, and capability to reach a greater sample group within a short period. While non-probability sampling is known to limit generalizability, bias was mitigated in this study by making sure participation was opened to diverse demographic segments. Respondents were obtained from various occupational sectors and education levels to replicate a realistic cross-section of the fasting target population. To increase response accuracy, participants were notified that the study was observing anonymity by ensuring that no identifying information was collected. Lastly, the data collection phase occurred within the middle weeks of Ramadan; this was considered appropriate since fasting routines had been fully established to ensure that the obtained responses would accurately reflect characteristic behaviors during the period.

### 2.5 Demographic characteristics

The target demographic was predominantly middle-aged participants who were educated. The age brackets were bundled in groups of 10 years, starting from 18 years. Data on education levels were collected to denote the highest level of schooling, employment status signified the current form of work engagement, nature of work illustrated the time of work engagement, and the number of shifts completed, while health status exhibited the well-being of the participants. This demographic profile was considered since it suggested that they were well-informed, professionally active, and likely to possess moderate-to-high health literacy; it created an ideal base for examining awareness and behavioral distinctions associated with oral health during Ramadan.

### 2.6 Data collection

The data collection phase was implemented through a structured, self-administered online questionnaire that was created specifically for this study. The survey aimed at capturing quantitative data on oral health knowledge, practices, attitudes, and sugar consumption during the Ramadan period. It was made to be accessible on both computers and mobile devices, which ensured convenience and inclusivity when completing it. The questionnaire contained several sections that captured various aspects of oral health knowledge, practices, attitudes, and sugar consumption. It included:

- **Sociodemographic data** – It captured background details like age, gender, education, occupation, work schedule, and health status.
- **Oral Hygiene Behavior** – It recorded usage of toothbrush, toothpaste, floss, and mouthwash before and during Ramadan, as well as assessing frequency of dental visits.
- **Dietary Habits** – Put emphasis on foods consumed at Iftar, Suhoor, and after Taraweeh, focusing on sweetened foods, dates, and beverages.
- **Knowledge Assessment** – Examined the beliefs concerning dental procedures and fasting validity depending on Islamic rulings and dental science.
- **Attitude Evaluation** – Explored perceptions of dental care during fasting, health authority awareness, and reception of preventive dental visits.
- **Behavioral Control** – Scrutinized respondents’ control over personal and children's sugar intake during Ramadan.

The questionnaire was pilot tested with a small group of people to guarantee it had clarity, reliability, and cultural appropriateness. The feedback obtained from the respondents in the pilot test was used to guide minor revisions before final distribution to the target sample.

### 2.7 Variable construction

Quantification of complex constructs was done by generating composite indices, which comprised:

- **Knowledge Score (KS):** These are seven belief-based questions that were scored 1 for correct responses and 0 for incorrect or uncertain ones; they produced a total score from 0 to 7.
- **Attitude Score (AS):** There are four items that reflect preventive orientation, belief in awareness raised by authorities, and timing preferences categorizing favorable or unfavorable attitudes.
- **Sugar Consumption Score (SCS):** Every sweet-related dietary selection received one point across three meal categories (Iftar, Suhoor, and post-Taraweeh), yielding a range of 0–3.

### 2.8 Data cleaning and validation

Given that data cleaning is an important element in the research process, the collected data underwent multiple stages of verification. Initial screening was done to eliminate duplicate submissions, blank responses, and logically inconsistent answers. Missing values in the data were minimal and were addressed via listwise deletion. Also, numeric coding was executed for all categorical variables in a manner that facilitated statistical testing. Lastly, outliers were assessed but were retained if considered reasonable. The descriptive summaries and frequency checks applied to the dataset confirmed internal consistency before commencing on analytical testing (16).

### 2.9 Statistical analysis

Data analysis for the cleaned data was conducted using IBM SPSS Statistics, a quantitative analytical software. The analytical strategy was a combination of descriptive, inferential, and modeling techniques seeking to comprehensively address the objectives and hypotheses of this study. Descriptive statistics entailed the determination of frequencies, means, and percentages for the summarization of demographic variables, oral health behaviors, and dietary patterns (17).

Inferential analyses were considered to examine relationships and differences as outlined below:

- **Chi-square (*χ*^2^)** tests were used to evaluate categorical associations like education level and brushing beliefs, employment and perceived awareness, and age group and dental treatment perceptions.
- **Kruskal–Wallis tests** were utilized for comparison of nonparametric distributions of knowledge and sugar scores across categorical variables like education, age, and employment.
- **McNemar's test** was used to assess pre- and post-Ramadan differences in brushing behavior; this tested whether fasting influenced oral hygiene practices.
- **K-means cluster analysis** was employed to identify latent attitude patterns and segmented participants into ‘Proactive’ and ‘Cautious’ groups depending on their attitude profiles.
- **Spearman's correlation** examined ordinal relationships between education and knowledge scores.
- **Binary logistic regression** was used to test the KAB framework through modeling of brushing behavior as a dependent variable, while knowledge and attitude scores were considered as independent variables.

It is important to note that a significant level of *p* < 0.05 was implemented for all hypothesis tests. Statistical interpretation highlighted both significance and practical meaning by odds ratios and directionality of relationships. The findings obtained would be used to reveal the strength of associations between education and knowledge, employment and awareness, and age and dental anesthesia perception (17). Additionally, the logistic regression was utilized to indicate the strength of attitude in predictive influence on brushing behavior, rather than when knowledge was considered alone. Together, these analyses were used to validate the partial applicability of the Knowledge, Attitude, Behavior model, to indicate the importance of awareness while stressing that the transformation of knowledge into actionable behaviors can be mediated by favorable attitudes and cultural understanding (18).

Moreover, the statistical analysis will yield findings on sugar consumption to stress the impact of culture and tradition when making dietary choices during Ramadan. The obtained results will be expected to be in alignment with previous evidence (19), which reported that age-related patterns of sugar consumption had been implemented in other Middle Eastern and Asian contexts. Due to the availability of sugary foods in social gatherings and cultural festivities, data analysis in this study accentuates the challenges of encouraging dietary moderation during celebration and communal bonding events.

Additionally, the persistence of the existing misconceptions among families is another indication that there exists a knowledge gap; this transcends general education to ensure it overlaps into religious literacy. Although some participants might correctly believe that the usage of toothpaste may not invalidate fasting, there might be a considerable proportion of participants who might think otherwise or show uncertainty on the matter. Findings obtained from previous research (20) noted that a substantial proportion of adults across Saudi Arabia showed hesitation to use mouthwash or toothpaste when fasting because of the fear of breaking their fast. Such beliefs can be cited to increase the complexity of integrating religious and scientific comprehension. Hence, statistical analysis seeks to determine if there is a need to emphasize the essence of embracing culturally tailored educational programs that are harmonized with faith-based teachings for proper adherence to oral health science and religious practices.

Hence, this current study seeks to perform statistical tests that provide a comprehensive understanding of oral health knowledge, behaviors, and attitudes by exploring sugar consumption patterns among people living in Qatar during the Ramadan period. The statistical tests will consider the objectives of this study to obtain findings that bridge the gap between oral health education and culturally grounded behavioral understanding, and to obtain evidence-based recommendations that can support oral health promotion related to fasting practices. Multivariate analytical approaches, including logistic regression and behavioral clustering, were applied to examine simultaneous relationships among knowledge, attitudes, and behaviors.

## 3 Results

The demographic data showed that 199 participants took part in the study, yielding a response rate of 79.6% from the expected sample population. The sample predominantly consisted of middle-aged adults representing diverse nationalities and occupational sectors. Most participants held bachelor's degrees and were employed, indicating a relatively stable socioeconomic profile. Table 1 presents a detailed summary of the demographic characteristics of the study population.

**Table 1:** Demographic characteristics.

Attributes	Categories	Frequency	Percentages
Age	Less than 18	3	1.5
	18–28	22	11.1
	29–39	31	15.6
	40–50	89	44.7
	Above 50	54	27.1
Education levels	Illiterate (No education)	1	0.5
	Still student (school/university)	6	3
	Secondary completed	21	10.6
	Bachelor	95	47.7
	Diploma	13	6.5
	Higher degree	63	31.7
Employment status	Unemployed	9	4.5
	Self Employed	17	8.5
	A private Sector	20	10.1
	Governmental Entity	153	76.9
Nature of works	Not working	9	4.5
	Evening only	2	1
	Morning only	133	66.8
	Two shifts	44	22.1
	Three shifts	11	5.5
Health status	Healthy	160	80.4
	Diabetes, Hypertension, High cholesterol, heart disease	12	6
	Other diseases (Kidney disease, Anemia, Cancer)	27	13.6

From Table 1, it is evident that the study sample was predominantly composed of middle-aged and older adults, with more than two-thirds aged 40 years and above, reflecting a mature demographic profile. Educational attainment was high, as nearly four-fifths of respondents held at least a bachelor's degree, indicating a well-educated cohort. The majority were employed in governmental entities and worked, primarily on morning shifts, suggesting stable occupational and socioeconomic conditions. In terms of health, most participants reported being generally healthy, with a minority indicating chronic or other medical conditions. Overall, this demographic structure represents a relatively educated, employed, and health-conscious population, which may positively influence their awareness, attitudes, and practices related to oral health during Ramadan. Participants were first asked about their frequency of visiting the dentist for a check-up. The results indicate that most respondents visit the dentist only in emergencies, highlighting a predominantly reactive rather than preventive approach to oral healthcare. Fewer participants reported attending checkups every 3–6 months or every 1–2 years, which are intervals generally recommended for maintaining oral health. This pattern suggests limited adherence to regular preventive dental visits and reflects a possible gap in awareness or prioritization of routine oral examinations. The findings underscore the need for enhanced health education initiatives emphasizing the importance of periodic dental checkups as a means of early detection and prevention of oral diseases, particularly within community-based oral health promotion programs.

Participants were asked what they typically consumed to break their fast (Iftar), and the results showed that the majority of respondents reported breaking their fast with dates, either alone or in combination with water, aligning closely with traditional and religious practices recommended for Iftar. A smaller proportion preferred water alone or dates accompanied by sweetened juices, while very few opted for only juices or Arabic coffee. This pattern reflects a culturally consistent and nutritionally balanced fasting habit, as dates provide a quick source of natural sugars and essential nutrients to restore energy after fasting. However, the relatively low preference for plain water and the consumption of sweetened beverages among some participants highlight an opportunity for targeted dietary education promoting healthier hydration and reduced sugar intake during Ramadan.

An enquiry on what the participants preferred to eat after Taraweeh prayer revealed that most respondents reported preferring a plate of sweets after the Taraweeh prayer, followed by those who indicated eating nothing during that period. Traditional dishes and light options, such as fruit and salad, were chosen in smaller proportions, while only a minority preferred healthy meals or fast food. These findings suggest that post-Taraweeh eating habits tend to favor sweet and calorie-dense foods, which may contribute to excessive sugar intake during Ramadan evenings. The limited consumption of balanced or nutrient-rich meals highlights the importance of public health education promoting healthier post-prayer dietary choices, focusing on moderation of sweets and increased inclusion of fruits, vegetables, and lighter meals to support overall well-being and oral health during the fasting month.

**Figure 1:**
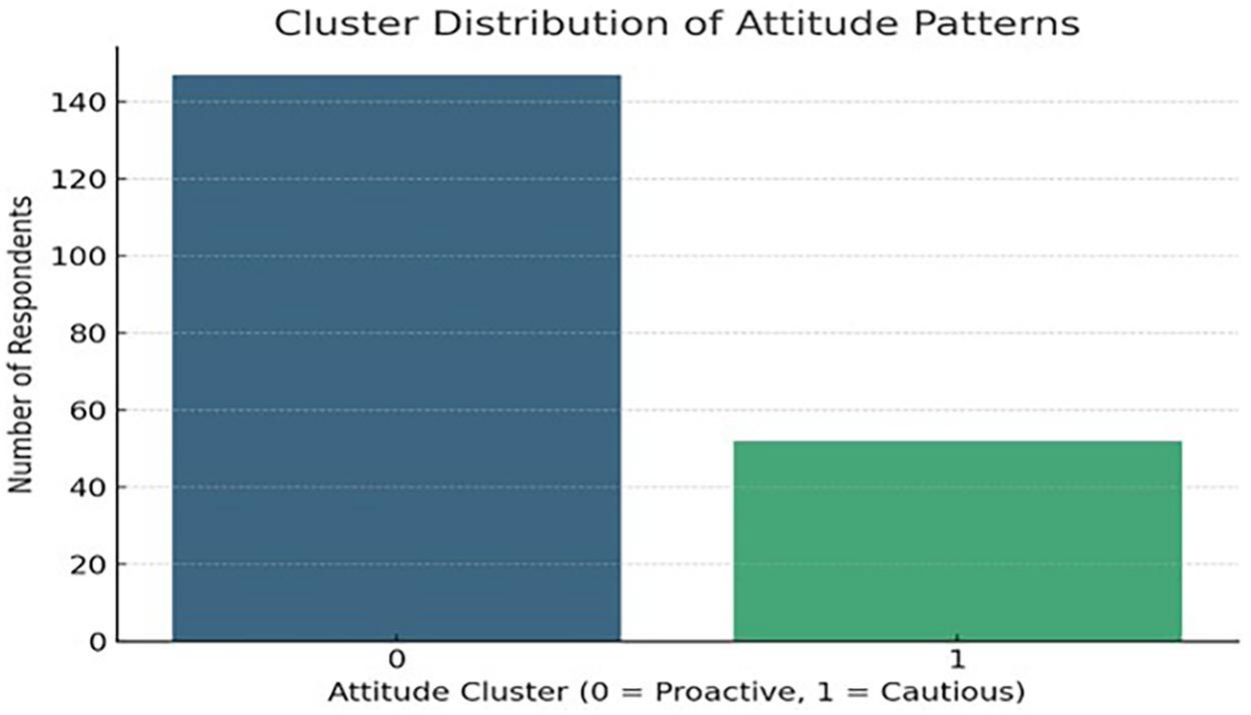
Cluster distribution of attitude patterns.

Participants were questioned about what they eat for Suhoor, the results are shown in Figure 1.

The majority of participants reported consuming a healthy snack at Suhoor, followed by those who ate leftover Iftar meals. A smaller proportion relied solely on water or consumed dates and yogurt, while very few reported eating nothing or having meals with high fat content and added sweets. These findings indicate that most respondents demonstrate a relatively balanced and health-conscious approach to the pre-dawn meal, reflecting an understanding of the importance of nutritious intake before fasting. However, the presence of a subgroup consuming calorie-dense or minimal Suhoor options underscores the need for continued nutritional awareness campaigns promoting light, high-fiber, and hydrating foods at Suhoor to sustain energy levels and support oral and overall health throughout the fasting hours.

**Figure 2:**
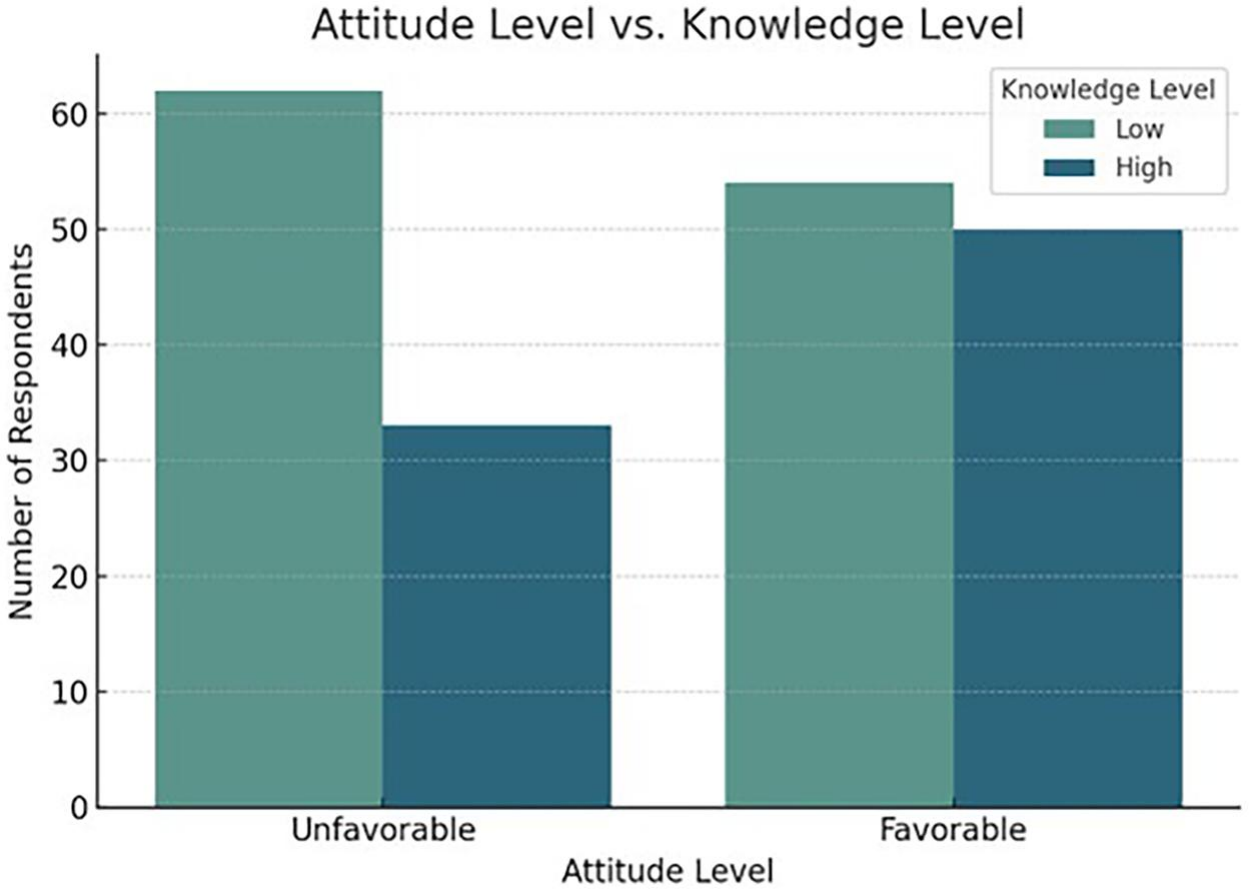
Attitude Level vs. Knowledge Level.

Participants were asked whether they controlled their children’s consumption of sweets during Ramadan, and the results are shown in Figure 2.

The majority of respondents (nearly two-thirds) reported actively controlling both their own and their children's consumption of sweets during Ramadan, reflecting a high level of dietary awareness within families. A smaller proportion admitted to not exercising control, while a minimal group indicated that they personally indulge but restrict their children's intake. This distribution suggests that most participants demonstrate responsible and health-conscious behaviors regarding sugar consumption, likely influenced by growing public awareness of the health risks associated with excessive sugar intake. Nonetheless, the presence of individuals who do not regulate their dietary habits underscores the need for ongoing health education programs that emphasize the importance of balanced sugar consumption for both adults and children, particularly during festive periods such as Ramadan.

**Figure 3:**
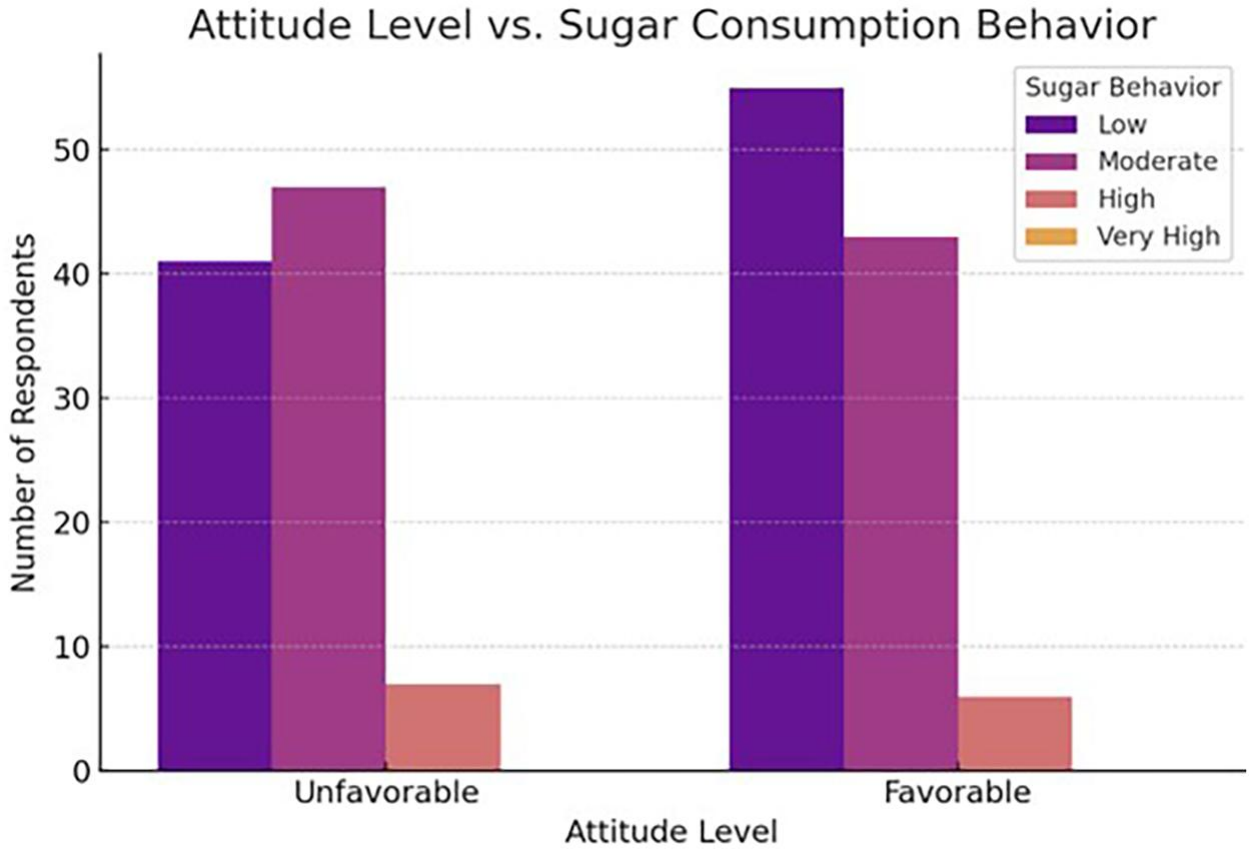
Attitude level vs. Sugar Consumption Behavior.

Participation in the traditional Qatari event or similar celebrations was inquired and the results are shown in Figure 3.

The findings reveal that a substantial majority of respondents (73.4%) reported participating in the traditional Qatari event ‘Garangao’ or similar celebrations, while only 26.6% indicated non-participation. This high level of engagement underscores the continued cultural significance and social integration of Garangao within the community, particularly as a family-centered activity promoting social cohesion and intergenerational participation during Ramadan. Such widespread involvement provides an effective cultural platform for public health outreach, suggesting that community-based events like Garangao could be strategically utilized to promote oral health awareness and healthy dietary behaviors among children and families in culturally resonant ways.

Participants were asked whether traditional events include distributing various types of sweets, the results revealed that an overwhelming majority of participants (97.0%) reported that their traditional events, such as Garangao, include the distribution of various types of sweets, while only 3.0% indicated otherwise. This finding underscores the profound cultural connection between traditional celebrations and sweet consumption in Qatari society. While such practices reinforce social bonds and cultural identity, they also contribute to increased sugar exposure, particularly among children. Therefore, these occasions present valuable opportunities for targeted oral health promotion and public awareness campaigns, emphasizing moderation in sweet consumption and encouraging the inclusion of healthier, low-sugar alternatives during cultural festivities to balance tradition with health-conscious practices.

An enquiry among the participants was done on whether their oral routine had changed during Ramadan compared to regular days, the results noted that most respondents (82.4%) reported no change in their oral care routine during Ramadan, whereas only 17.6% indicated that their habits had changed compared to regular days. This suggests that most participants maintained consistent oral hygiene behaviors despite the altered daily schedule associated with fasting. However, the minority reporting changes may reflect individuals who reduced brushing frequency or modified their timing due to religious concerns or misconceptions about the permissibility of oral care during fasting hours. These findings underscore the need for educational initiatives led by health authorities and religious scholars to clarify acceptable oral hygiene practices during Ramadan, thereby supporting continuous and effective oral care throughout the fasting month.

Participants were asked what they used to brush their teeth with before Ramadan, and the results revealed that an overwhelming majority of respondents (95.5%) reported using a toothbrush and toothpaste to clean their teeth before Ramadan, indicating strong adherence to conventional oral hygiene practices. A very small proportion used dental floss (2.0%), electric toothbrushes (1.0%), or traditional alternatives such as miswak (0.5%), while only 0.5% reported not brushing their teeth. This distribution reflects a high level of oral hygiene awareness and a preference for modern tooth-cleaning tools over traditional methods. Nonetheless, the minimal use of adjunctive cleaning aids, such as floss or interdental devices, highlights an area for improvement, emphasizing the importance of promoting comprehensive oral hygiene routines that include interdental cleaning and tongue care, alongside regular toothbrushing, for optimal oral health maintenance.

Participants were also queried on what they used to brush their teeth with during the Ramadan period, and the results showed that during Ramadan, most respondents (93.0%) continued to use a toothbrush and toothpaste for oral hygiene, indicating a strong commitment to maintaining regular dental care practices despite fasting. A small increase was observed in the use of dental floss (3.5%) and interdental cleaning tools (1.5%), while the use of electric toothbrushes and miswak (1.0%) remained minimal. Compared with pre-Ramadan habits, these findings suggest only a slight decline in toothpaste-based brushing, possibly reflecting hesitation or misconceptions regarding its permissibility during fasting hours. Overall, the results demonstrate a high level of oral hygiene awareness; however, continuous educational efforts are needed to clarify religious guidance on oral care during fasting and to encourage complementary practices, such as flossing and tongue cleaning, for comprehensive oral health maintenance.

Participants were questioned on whether using a toothbrush with toothpaste affected their fasting, results revealed that many respondents (69.3%) correctly believed that using a toothbrush with toothpaste does not invalidate fasting, whereas 23.1% held the misconception that it does, and 7.5% were uncertain. This distribution reflects a generally good level of awareness regarding oral hygiene practices during fasting, though a considerable proportion still harbors religious or cultural misconceptions. The persistence of such uncertainty indicates the need for collaborative educational efforts between dental professionals and religious authorities to provide clear guidance on permissible oral care practices during Ramadan. Strengthening this understanding can help promote consistent oral hygiene behaviors without fear of compromising religious observance.

An enquiry among the participants was done to determine their perspective on gum treatment and teeth cleaning during daytime in Ramadan, the obtained results noted that more than half of the respondents (58.8%) believed that gum treatment and teeth cleaning during the daytime in Ramadan do not break the fast, whereas 12.1% thought such procedures would invalidate fasting, and 29.1% were uncertain. These results indicate a generally positive awareness of the permissibility of routine dental care during fasting, yet a significant portion of participants still exhibit uncertainty or misconceptions regarding religious rulings. The findings highlight the importance of integrating faith-based oral health education, whereby religious authorities and dental professionals collaborate to clarify that professional dental cleaning and gum treatment, when performed cautiously, do not compromise fasting. This approach could improve public confidence in seeking necessary dental care during Ramadan while maintaining religious observance.

Additionally, participants were asked about their perspective on dental fillings during daytime in Ramadan, the obtained results observed that most respondents (61.8%) believed that dental fillings performed during the daytime in Ramadan do not break the fast, while 11.1% thought such procedures would invalidate fasting, and 27.1% expressed uncertainty. These results indicate a generally favorable level of awareness among participants regarding the permissibility of dental restorative treatments during fasting hours. However, the presence of a notable proportion of individuals holding misconceptions or uncertainty underscores the need for religiously aligned oral health education. Collaborative efforts between dental professionals and religious scholars can help dispel doubts, ensuring that individuals feel confident in seeking necessary dental care during Ramadan without fear of violating fasting obligations.

Participants were asked about what they think regarding dental anesthesia during the daytime in Ramadan, and the results showed that more than half of the respondents (57.3%) correctly believed that administering dental anesthesia during the daytime in Ramadan does not break the fast, while 13.1% thought it would invalidate fasting, and 29.6% were uncertain. These findings suggest a generally positive understanding of dental anesthesia practices among fasting individuals, yet a considerable degree of uncertainty remains. Misconceptions may arise from confusion about whether injectable treatments are permissible during fasting. Therefore, these results highlight the importance of interdisciplinary communication between dental practitioners and religious authorities in providing clear, culturally sensitive explanations of permissible dental interventions. Such educational reinforcement can help reduce anxiety about seeking dental care during fasting hours and promote timely treatment without compromising religious observance.

An enquiry on participants’ thoughts on what they think about root canal treatment during the daytime in Ramadan, the results showed that more than half of the respondents (55.3%) believed that undergoing root canal treatment during the daytime in Ramadan does not break the fast, while 13.1% thought it would, and 31.7% were uncertain. These results indicate that the majority possess a correct understanding aligned with religious guidance, which considers such dental procedures permissible as long as no substances are swallowed. However, the relatively high proportion of participants expressing uncertainty highlights ongoing ambiguity regarding invasive dental treatments during fasting hours. Strengthening collaboration between dental professionals and religious scholars is therefore essential to clarify misconceptions and reassure patients that necessary therapeutic procedures, such as root canal treatments, can be safely performed during fasting without compromising religious observance.

Participants were asked about what they think concerning tooth extraction during the daytime in Ramadan, and the results showed that less than half of the respondents (44.7%) believed that tooth extraction during the daytime in Ramadan does not break the fast, while 23.6% thought it does, and 31.7% were uncertain. This indicates a moderate level of awareness, yet a substantial proportion of participants remain unclear or misinformed about the permissibility of dental surgical procedures during fasting hours. The higher level of hesitation compared to other dental treatments may reflect fears of bleeding, ingestion, or discomfort following extraction, which can raise religious concerns. These findings underscore the need for targeted health education initiatives that integrate religious guidance and professional dental advice, clarifying that tooth extractions, when conducted carefully, do not invalidate fasting. This approach encourages timely treatment and prevents unnecessary delays in essential dental care during Ramadan.

An enquiry among the participants was done to determine their perspective on taking dental impressions during daytime in Ramadan, the obtained results showed that most participants (68.3%) believed that taking dental impressions during the daytime in Ramadan does not break the fast, while 6.5% thought it would, and 25.1% were uncertain. This high level of correct understanding reflects growing awareness that non-invasive dental procedures, such as impressions, are permissible during fasting when precautions are taken to avoid swallowing materials. However, the persistence of uncertainty among a quarter of respondents highlights the need for further community education to clarify the distinction between diagnostic or procedural interventions and actions that may invalidate fasting. Enhanced collaboration between religious authorities and dental practitioners can help reinforce accurate knowledge, reduce misconceptions, and promote timely access to routine dental care during Ramadan without compromising religious obligations.

Participants were asked about dental prosthesis treatment during the daytime in Ramadan, the results showed that more than half of the respondents (56.3%) correctly believed that dental prosthesis treatment during the daytime in Ramadan does not break the fast, while 13.1% thought it would invalidate fasting, and 30.7% were uncertain. These findings indicate a generally good level of awareness regarding the permissibility of prosthodontic procedures, such as fitting or adjusting dentures, during fasting hours. However, the considerable proportion of participants who were unsure or held misconceptions highlights lingering concerns about the potential ingestion of materials or adhesives, as well as religious and procedural concerns. Targeted educational initiatives that involve both dental professionals and religious scholars are recommended to reinforce accurate understanding, helping patients feel confident in pursuing routine prosthodontic care during Ramadan without compromising their fasting obligations.

Moreover, participants were asked about their perspective on swallowing water during dental treatment while fasting, the obtained results showed that the majority of respondents (83.4%) believed that a fasting individual might swallow water during dental treatment, whereas only 16.6% thought otherwise. This perception reflects a widespread concern about the risk of unintentionally breaking the fast when undergoing dental procedures, which may contribute to patients’ reluctance to seek dental care during Ramadan. The finding underscores the need for clear guidance and reassurance from both dental professionals and religious authorities, emphasizing that with appropriate precautions—such as the use of suction and controlled water flow; dental treatment can be safely performed without invalidating the fast. Addressing this misconception through public awareness and professional communication can help promote timely and uninterrupted oral healthcare during the fasting month.

It was enquired about their perspective on awareness from health authorities during Ramadan, and the results showed that more than half of the participants (52.8%) reported insufficient awareness from health authorities about oral and dental health during Ramadan, while only 18.1% believed that such awareness exists, and 29.1% were uncertain. These results point to a significant communication gap in public health education regarding oral care during the fasting month. The lack of visible outreach and consistent messaging may contribute to the persistence of misconceptions about dental treatment and fasting. Therefore, the findings emphasize the need for structured, culturally tailored health promotion campaigns led by national health authorities, in collaboration with dental professionals and religious leaders, to enhance public understanding and encourage preventive oral health practices throughout Ramadan.

**Figure 4:**
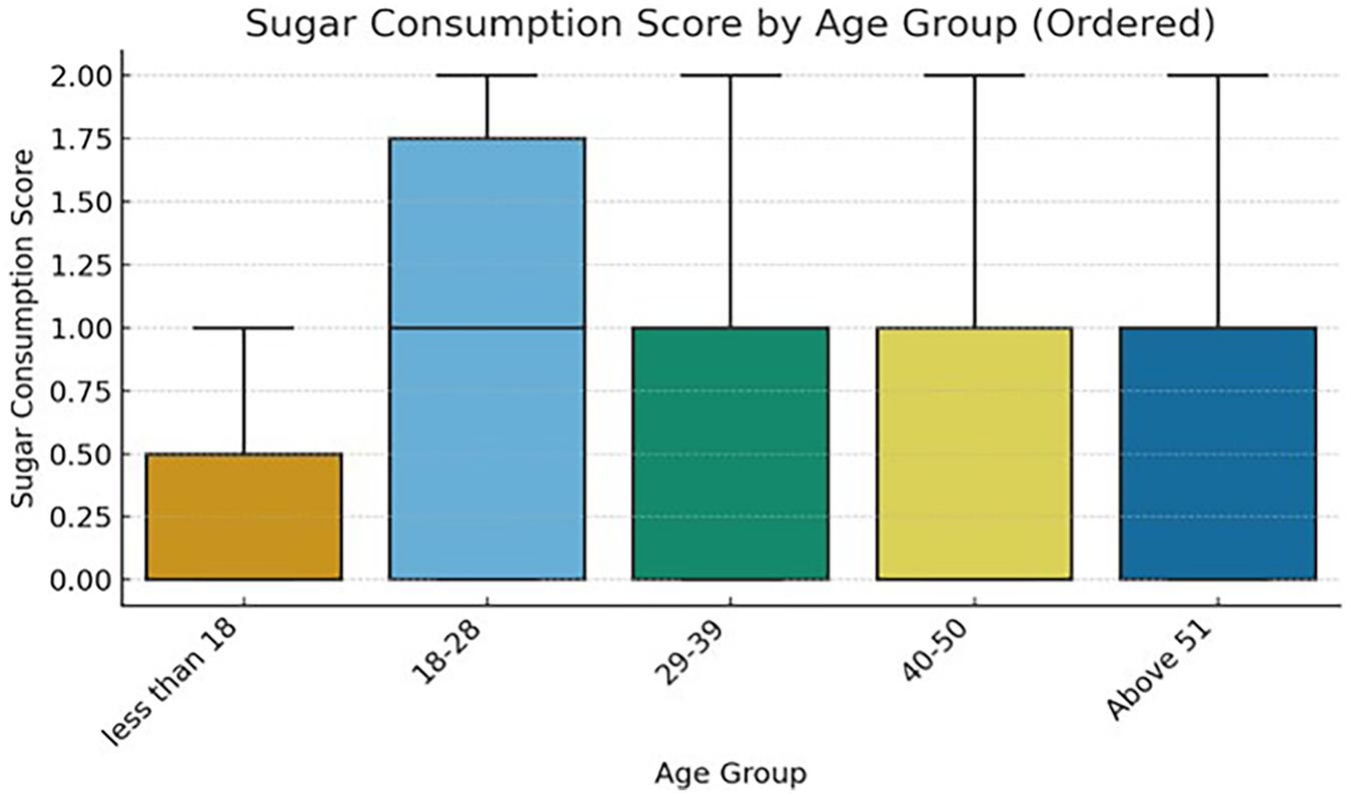
Sugar consumption score by Age group.

Participants were asked about preferred methods to deliver health information, and the results are shown in Figure 4.

Many respondents (54.8%) preferred official social media platforms managed by health authorities as the primary channel for receiving health information, followed by messages communicated through medical personnel quoting official sources (18.6%). Traditional media, such as television (10.6%) and public social media outlets (9.5%), were less favored, while SMS (5.0%) and celebrity- or influencer-based communication (1.5%) ranked lowest. These findings highlight a strong trust in institutional and authoritative sources, reflecting the community's preference for credible, evidence-based health communication. The results highlight the importance of utilizing digital platforms managed by official health organizations to effectively disseminate oral and dental health messages, ensuring both cultural relevance and scientific accuracy in public health campaigns during Ramadan and beyond.

### 3.1 Cross-tabulations and Chi-square tests

Inferential analyses were considered to examine the relationships and differences among the variables used in this study. The relationship between education level and brushing during fasting was determined using Chi-Square, as shown in Table 2.

**Table 2:** Education level and brushing during fastin*g.*

Statistic	Value
Chi-square (*χ*^2^)	22.74
Degrees of freedom (df)	10
*p*-value	0.0118

It was observed that there is a statistically significant association (*p* < 0.05) between education level and the belief that using a toothbrush with toothpaste breaks a fast. Respondents with higher education were more likely to answer ‘*No’* to ‘brushing does not invalidate fasting’ compared to those with lower education levels, since they showed greater uncertainty (*‘I don't know’*).

Another Chi-square test was done to explore the relationship between Employment Status by Awareness from Health Authorities, the results noted that there is a significant relationship between the employment sector and perceived awareness efforts from health authorities during Ramadan. Government employees were more likely to believe there is *insufficient awareness*, whereas private-sector and self-employed respondents were more inclined to respond, ‘*I don't know’*. This suggests that awareness campaigns may not be reaching all occupational groups equally.

In addition, another Chi-square test was executed to determine the relationship between Age Group and Opinion on Dental Anesthesia During Fasting, the results are shown in Table 3.

**Table 3:** Age group by opinion on dental anesthesia during fastin*g.*

Statistic	Value
Chi-square (χ^2^)	16.79
Degrees of freedom (df)	8
*p*-value	0.0324

The findings in Table 3 revealed that a significant difference exists across age groups in their perceptions of dental anesthesia during fasting. Participants aged 40–50 were most confident that anesthesia *does not break the fast,* while younger respondents (18–28 years) demonstrated the highest level of uncertainty. This indicates a knowledge gap between age cohorts, underscoring the need for age-specific educational materials.

A comparison of the Chi-square test findings was done, including the interpretation of what the relationship of the variables meant, the results showed that all three tested relationships were statistically significant (*p* < 0.05), Education and employment appear as major predictors of awareness and misconceptions regarding oral health practices during Ramadan. Deeper exploration revealed that middle-aged and highly educated respondents demonstrated a better understanding and greater confidence in distinguishing between permissible and impermissible dental procedures.

#### 3.1.1 Sugar consumption behavior analysis

This assessment sought to identify all sweet-related responses from Iftar, Suhoor, and post-Taraweeh questions. A ’Sugar Consumption Score’ was constructed by assigning 1 point for each sweet-related choice. The sugar scores were analyzed against age, education, and employment using Chi-square or Kruskal–Wallis tests.

##### 3.1.1.1 Construction of the sugar consumption score (SCS)

Each sweet-related choice (e.g., *sweetened juices, desserts, pastries, fast food, sweets*) was assigned 1 point. Scores were then summed across the three meals:

SugarScore=Iftar(sweet)+Taraweeh(sweet)+Suhoor(sweet)

The findings of Chi-square tests on the relationship between sugar scores and age, education, or employment are displayed in Table 4.

**Table 4:** Sugar consumption score (SCS*).*

Demographic factor	Statistical finding	Visual insight
Age	Significant (χ^2^, *p* = 0.008)	Younger respondents consume more sweets
Education	Significant (χ^2^, *p* = 0.003)	Lower education linked to higher sweet intake
Employment	Not significant (χ^2^, *p* = 0.311)	Minimal impact on sugar patterns

Chi-square results indicate significant associations between sugar consumption and both age and education, suggesting categorical differences in sweet-eating tendencies among specific groups (e.g., younger or less-educated participants showing higher sweet preference).

### 3.2 Interpretation and insights

The findings revealed that there was moderate consumption of sweets, the mean sugar score of 0.58 suggests that most respondents consume sweet foodstuff occasionally, rather than habitually. Examining the demographic patterns showed that younger age groups and those with lower education levels exhibit a higher frequency of sweet food choices (as supported by *χ*^2^ significance). Also, employment type does not significantly influence sugar behavior. Hence, the potential public health implications of these findings are that there is a need for awareness campaigns targeting younger populations about the risks associated with consuming excessive sweetened drinks and desserts during Ramadan, in addition to encouraging substitution with fruits, nuts, and traditional low-sugar options.

The relationship between Sugar Consumption Score and Age was examined and is shown in Figure 4. It was observed that most age groups exhibit low-to-moderate sweet intake, centered around a Sugar Score of 0–1. Younger participants (<30 years) exhibit slightly higher variability, with more frequent consumption of sweet foods, while older groups (≥50 years) have lower median scores, reflecting reduced sweet intake.

**Figure 5:**
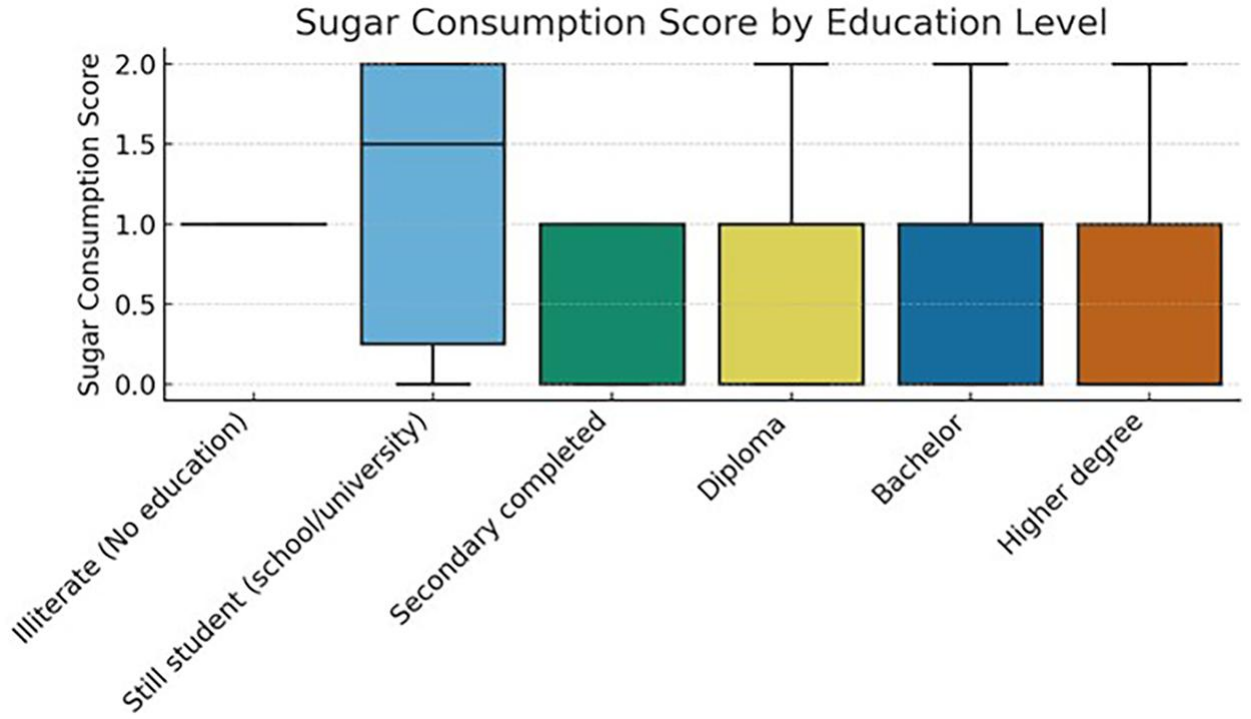
Sugar consumption score by education level.

The relationship between Sugar Consumption Score and Education Level was examined and is shown in Figure 5. The findings revealed that sweet intake declines progressively with higher education. Students and secondary graduates report more frequent sweet consumption, while respondents with bachelor's and postgraduate degrees report lower median scores.

#### 3.2.1 Knowledge assessment

This assessment used questions about dental procedures and beliefs to create a ‘Knowledge Score’ by assigning 1 point for each correct response based on dental and religious guidance. Analysis was appropriately done on knowledge score differences across education, age, and employment using Kruskal–Wallis tests.

##### 3.2.1.1 Knowledge variables

Seven items from the survey were analyzed to assess beliefs about *gum treatment, dental fillings, anesthesia, root canal treatment, extraction, impressions,* and *prosthesis placement* during fasting. The scoring system was that each correct response (“It does not break the fast”) was assigned 1 point, while Knowledge Score (KS) was the sum of correct answers across the 7 items.

0≤KS≤7

On average, respondents correctly answered 4 out of 7 questions, a substantial proportion scored either very low or perfect (0 or 7), suggesting polarized knowledge levels — some were fully aware, while others were uncertain or misinformed.

Kruskal–Wallis Test, which is a nonparametric ANOVA, was done, and the results are presented in Table 5.

**Table 5:** Kruskal–wallis test (nonparametric ANOVA*).*

Variable	H statistic	*p*-value	Significance
Age group	6.05	0.195	Not significant
Education level	25.52	0.00011	Significant
Employment status	2.94	0.401	Not significant

**Figure 6:**
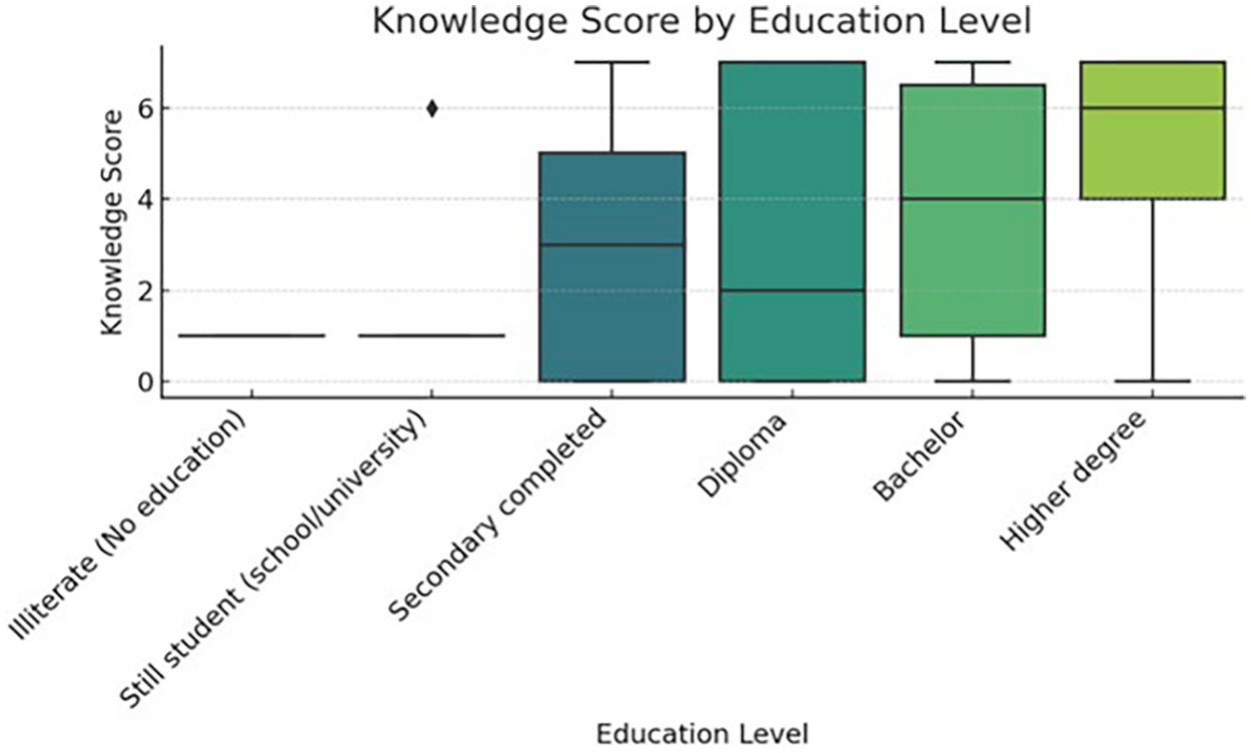
Knowledge score by education level.

The interpretation of these results is that Education Level has a strong, statistically significant effect on knowledge (*p* < 0.001). Knowledge scores increase with higher education, confirming education as a key determinant of dental awareness. However, Age and Employment Status do not significantly influence knowledge scores. Respondents with higher education (bachelor's and postgraduate degrees) demonstrate a markedly better awareness of fasting-related dental rulings. Misconceptions persist among less educated and younger individuals, particularly regarding invasive treatments (e.g., extractions, root canals). Hence, targeted public health education, especially through mosques, universities, and health authorities, can be considered to bridge this gap by clarifying permissible dental practices during Ramadan. Knowledge Score against Education Level is illustrated in Figure 6.

#### 3.2.2 Attitude assessment

This assessment summarized responses about dental visits and beliefs during Ramadan. Group respondents were categorized into favorable vs. unfavorable attitudes. Additionally, cluster analysis was performed to find patterns (if feasible) and an analysis of attitudes in relation to knowledge and behavior. To evaluate attitudes toward dental care during Ramadan, respondents were classified into favorable or unfavorable groups to examine how these attitudes relate to knowledge and sugar consumption behavior. The four survey items were analyzed as indicators of dental attitude, namely ‘How often should you visit the dentist for a checkup?’

*(Measures dental responsibility and preventive behavior), ‘*When is it preferable to visit the dental clinic during Ramadan?’ *(Reflects acceptance of dental visits during fasting), ‘*Is there sufficient awareness from health authorities about oral and dental health during Ramadan?’ *(Perceived institutional support), and ‘*Does using a toothbrush with toothpaste affect fasting?’ *(Religious and behavioral acceptance of oral hygiene).* Using the median score (1.25), participants were divided as follows: Favorable Attitude Group (≥ median) at 52% while Unfavorable Attitude Group (< median) was 48%. These classifications reflect a near-even split between proactive and cautious participants regarding dental care during fasting.

### 3.3 Cluster analysis (behavioral segmentation)

A K-means cluster analysis (k = 2) was performed on the encoded attitude variables to explore latent patterns. The analysis revealed two groups: Cluster 0, a ‘Confident and Proactive’ group with higher visit frequency and acceptance of dental care during fasting, and Cluster 1, a ‘Cautious and Traditional’ group that avoids treatment during fasting and shows uncertainty about toothpaste permissibility.

#### 3.3.1 Relationship between attitude, knowledge, and behavior

The analysis examined the relationship between Attitude and Knowledge, as well as between Attitude and Sugar Consumption Behavior. The results indicated a weak but near-significant trend showing that individuals with more favorable attitudes tend to have higher levels of knowledge. In contrast, sugar-related behavior showed no meaningful association with attitude, suggesting that sweet intake during Ramadan is influenced more by cultural traditions than by oral health awareness.

### 3.4 Key insights

The overall findings revealed that attitudes toward oral care during Ramadan were moderately favorable, with many participants expressing openness to receiving dental care while fasting. Two distinct behavioral orientations emerged, representing proactive individuals and those who were more cautious or traditional in their decisions. Knowledge was marginally linked to more positive attitudes, whereas sugar consumption behavior remained unaffected by attitude patterns. These insights suggest that improving knowledge could help shift hesitant participants toward more proactive behaviors, and that future awareness efforts should particularly address the concerns of cautious individuals by emphasizing religious permissibility and the importance of preventive dental care.

Cluster distribution of Attitude Patterns was done to classify the participants into two distinctive groups, as shown in Figure 1. The cluster distribution of Attitude Patterns identified two clusters that confirmed distinct respondent orientations, namely Cluster 0 (‘Proactive’) was for those comfortable with dental visits and hygiene during fasting, while Cluster 1 (‘Cautious’) was for those hesitant toward dental care during fasting. Both groups are nearly balanced, indicating a diverse spectrum of attitudes in the population.

Another cluster distribution of Attitude Level against Knowledge Level was done to classify the participants into two unique groups, as shown in Figure 2. Assessment of Attitude Level vs. Knowledge Level revealed that respondents with favorable attitudes show a noticeably higher proportion of high knowledge levels. This aligns with the borderline significant association (*p* ≈ 0.078), suggesting greater knowledge fosters more confident, health-positive attitudes.

Moreover, another cluster distribution of Attitude Level against Sugar Consumption Behavior was done to classify the participants into two unique groups, as shown in Figure 3.

Examination of Attitude Level against Sugar Consumption Behavior revealed that the distribution of sugar consumption categories (Low–Very High) remains nearly identical across attitude levels. This supports the statistical finding (*p* = 0.388) that dietary behavior is largely independent of attitude toward dental care or fasting beliefs.

Distribution of Attitude Scores

- The histogram demonstrates a slightly right-skewed distribution, indicating most participants maintain moderate to favorable attitudes.
- Very few respondents exhibit extremely unfavorable perspectives.

#### 3.4.1 Oral health behavior

This assessment compared responses on brushing and dental hygiene before and during Ramadan using McNemar's test for significant changes in paired data. Crosstab was used to compare behavior with beliefs (e.g., brushing vs. belief about toothpaste breaking the fast). To assess changes in toothbrushing and oral hygiene practices before and during Ramadan, and to evaluate whether brushing habits are associated with religious beliefs about fasting.

Survey Items Used included ‘*What do you use to brush your teeth before Ramadan?’*, ‘*What do you use to brush your teeth during Ramadan?’, and ‘Does using a toothbrush with toothpaste affect fasting?’.* Participants were coded as: 1 for ‘Brushes using a toothbrush/toothpaste’ and 0 for ‘Does not use toothbrush/toothpaste’.

##### 3.4.1.1 Mcnemar’s test (paired binary comparison)

The test revealed that there was no statistically significant change (*p* > 0.05) in brushing habits before and during Ramadan. Most respondents maintained consistent oral hygiene behavior throughout the fasting period, while a small subset (6 respondents) stopped brushing, possibly due to concerns about fasting validity.

##### 3.4.1.2 Relationship between brushing behavior and belief about toothpaste

The association between brushing behavior and belief about fasting is not statistically significant (*p* > 0.05). However, descriptively, those who believe toothpaste does not break the fast are more likely to maintain brushing (≈ 132 respondents).

#### 3.4.2 Summary of key findings

It was noted that the majority of participants demonstrate stable oral hygiene practices through Ramadan. Misconceptions persist among a minor subset who associate brushing with invalidating fasting. Public health messaging should emphasize that brushing with toothpaste does not break a fast, supporting both religious observance and dental health maintenance.

##### 3.4.2.1 Knowledge–attitude–behavior (KAB) model

This assessment sought to build a model showing Knowledge → Attitude → Behavior using path analysis or logistic regression to explore if knowledge predicts better attitudes and improved oral hygiene.

##### 3.4.2.2 Knowledge–attitude–behavior (KAB) logistic regression analysis

It was considered to assess whether knowledge and attitude predict oral hygiene behavior during Ramadan, a logistic regression model was estimated:

$$\mathrm{Logit}(P(\mathrm{Brush}\;\mathrm{During}\;\mathrm{Ramadan}))=\beta_{0}+\beta_{1}(\mathrm{Knowledge}\;\mathrm{Score})+\beta_{2}(\mathrm{Attitude}\;\mathrm{Score})$$

The findings of the KAB model are shown in Table 6. It was noted that Attitude plays a stronger predictive role than knowledge in determining oral hygiene behavior. Although marginal (*p* ≈ 0.11), the effect size is substantial (OR ≈ 3.2). Knowledge alone does not significantly predict behavior (*p* = 0.89), implying that awareness must translate into a positive attitude to influence practice. Thus, this aligns with the KAB theoretical pathway, suggesting an indirect (mediated) influence of knowledge on behavior through attitude.

**Table 6:** Model result*s.*

Predictor	Coefficient (*β*)	Std. error	*p*-value	Odds ratio (expβ)	Interpretation
Knowledge score	0.015	0.112	0.893	1.02	No significant direct effect of knowledge on brushing behavior.
Attitude score	1.166	0.735	0.113 (marginal)	3.21	Respondents with more favorable attitudes are ∼3 × more likely to maintain brushing during Ramadan.

It was observed that the Knowledge–Attitude–Behavior framework holds partially true in this population since Knowledge enhances Attitude, and Attitude substantially influences behavior, but knowledge by itself does not directly predict behavior. This suggests that educational interventions should focus not only on information dissemination but also on belief reinforcement and attitude shaping, particularly concerning fasting and oral care.

### 3.5 Hypothesis testing

The following five hypotheses were evaluated:

**H1:** People who believe toothpaste breaks the fast are less likely to brush during Ramadan.
**H2:** Higher education is associated with better knowledge about oral care during fasting.
**H3:** People who consume more sweets are less likely to control their children's intake.
**H4:** Knowledge of religious rulings predicts better oral hygiene behavior.
**H5:** Belief in sufficient health authority awareness is linked to better dental attitudes.

#### 3.5.1 H1: People who believe toothpaste breaks the fast are less likely to brush during ramadan

The test used is a 2 × 2 association (Belief = ‘Yes’ vs. ‘Not-Yes’) × Brushing during Ramadan (1/0). it showed that ‘Believe Yes’ had 42 who brushed and 4 who did not brush while ‘Believe No’ had 145 who brush and 8 who did not brush. The Fisher's exact *p*-value was 0.478, the *χ*^2^ was 0.608, while overall *p*-value was 0.58. This can be concluded that the relationship was not significant. Those who believe ‘breaks the fast’ *appear* less likely to brush, but the difference is not statistically supported.

#### 3.5.2 H2: Higher education is associated with better knowledge about oral care during fasting

The outcome of this test was Knowledge-Score (0–7) while the ordinal factor was Education level. Kruskal–Wallis's test reveals H = 25.52, *p* = 1.10 × 10⁻⁴, which means significant differences across education groups. The Spearman (ordinal education vs. knowledge) showed that correlation was 0.355, *p* = 2.60 × 10⁻⁷. This can be concluded to mean that there is strong evidence that higher education correlates with better knowledge.

#### 3.5.3 H3: People who consume more sweets are less likely to control their children’s intake

The variable considered was Sugar Score (0–3), while the control item was detected as ‘Do you control your own or your children's (if you have any) consumption of sweets during Ramadan?’ In this dataset, the detected responses yielded no affirmative ‘control’ cases under conservative mapping, so group comparison is not estimable (Mann–Whitney U and Spearman were both undefined). The conclusion made was that it was not testable with current coding, data needed to be recoded for testing to be executed.

#### 3.5.4 H4: Knowledge of religious rulings predicts better oral hygiene behavior

It utilized a Logistic regression model to determine the association of Brushing during Ramadan and Knowledge Score. The obtained coefficient was 0.016 (SE 0.111), *p* = 0.885, OR = 1.016. This can be interpreted to mean that there is no direct effect of knowledge on brushing behavior, this is consistent with the KAB results, where knowledge's influence is largely indirect via attitude.

#### 3.5.5 H5: Belief in sufficient health authority awareness is linked to better dental attitudes

The outcome variable was Attitude_Score (composite from attitude items) while Awareness belief (Yes/No/I don't know) was the factor variable. Kruskal–Wallis test revealed that H = 44.83, *p* = 1.84 × 10⁻^1^⁰, which shows a highly significant relationship. It can be concluded that those who believe awareness is sufficient exhibit meaningfully higher dental attitudes.

### 3.6 Conclusion of the results

The findings obtained from the analysis of the data collected in this study indicated that oral health awareness amongst individuals living in Qatar during Ramadan was generally high. However, there were notable gaps that persist in particular knowledge areas and attitudes that are influenced by cultural and religious beliefs. It was revealed that the majority of the participants were middle-aged, well-educated, and employed in governmental sectors; they reported maintaining consistent oral hygiene practices before, during, and after fasting to suggest a solid adherence to fundamental dental routines. However, some misconceptions were observed to persist, especially regarding the use of toothpaste and the acceptability of dental treatments within fasting hours; these might deter some individuals from seeking preventive medical care. Education was noted to have emerged as the strongest predictor of oral health knowledge, although attitudes tended to play a significant mediating role in influencing behavior. It was observed that participants with more favorable attitudes were three times more likely to uphold regular brushing behavior. Moreover, sugar consumption patterns echoed cultural traditions since younger and less-educated participants had higher intake of sweets, particularly after Taraweeh and during Garangao celebrations. This highlights the influence of social customs in shaping dietary behavior. Despite having widespread awareness of the importance of oral care, it was noted that over half of the respondents alleged that health authorities had not provided sufficient guidance during Ramadan. This underscores the existence of a communication gap between official governmental sources and the general public. Generally, the obtained results point out that while Qatar's fasting population exhibited commendable oral hygiene awareness, there was room for improvement in behavioral tendencies. This was dependent on strengthening culturally sensitive education, clarification of existing religious misconceptions, and promotion of balanced dietary practices that can be attained via collaborative efforts between religious leaders, educators, community leaders, and health professionals.

## 4 Discussion

This discussion interprets the principal findings of the study within the broader context of existing literature on oral health behavior during Ramadan and culturally influenced preventive health practices.

Several international studies have examined selected aspects of oral health during Ramadan, including halitosis, oral hygiene practices, and physiological changes related to fasting. However, research integrating behavioral, cultural, and dietary determinants of oral health within the lived context of Ramadan remains limited, particularly in the Qatari setting. To our knowledge, this study represents one of the first comprehensive investigations integrating oral health knowledge, attitudes, hygiene practices, and culturally influenced dietary behaviors during Ramadan within the Qatari population. By applying an integrated behavioral framework, the study provides new evidence on how religious beliefs, cultural practices, and health attitudes collectively shape preventive oral health behaviors during fasting and supports the development of culturally and faith-aligned oral health promotion strategies in Muslim communities.

### 4.1 Principal findings and interpretation

This study provides an integrated understanding of oral health knowledge, hygiene behaviors, attitudes, and sugar consumption patterns among adults living in Qatar during Ramadan. The findings indicate that although awareness regarding oral hygiene importance is generally high, behavioral practices remain influenced by religious misconceptions and culturally shaped dietary habits.

Similar behavioral patterns during fasting periods have been reported in previous regional and international research, where changes in daily routines were shown to influence both oral hygiene adherence and dietary practices (3, 5).

A key observation was the maintenance of routine oral hygiene practices among many participants despite fasting restrictions, suggesting that established preventive habits may persist even under altered daily schedules. Comparable findings have been documented among educated Gulf populations, where accessibility to dental services and health literacy supported continuity of oral hygiene behaviors (21). However, uncertainty regarding the permissibility of toothbrushing or mouthwash use during fasting remained evident, reinforcing earlier evidence demonstrating that religious interpretation continues to shape preventive health behavior in Muslim communities (4).

Educational attainment emerged as an important determinant of oral health knowledge, supporting extensive global literature linking education with improved health literacy and preventive behavior adoption (13, 14). Nevertheless, persistent uncertainty surrounding certain dental procedures indicates that formal education alone may not fully address culturally or religiously influenced health beliefs. These findings emphasize the importance of integrating scientifically accurate information with culturally sensitive communication strategies.

Perceptions of oral health promotion efforts also varied across occupational and demographic groups, suggesting uneven penetration of public health messaging. Previous studies have demonstrated that workplace-based and community-centered health initiatives significantly enhance preventive behavior adoption among adults (14). The present findings therefore highlight the need for more consistent dissemination of fasting-specific oral health guidance across diverse population sectors.

Age-related differences observed in attitudes toward dental care during fasting further support existing evidence indicating that health beliefs evolve with experience and healthcare exposure (19, 22). Younger individuals may rely more heavily on informal information sources, whereas older adults tend to demonstrate greater confidence in health decision-making. These patterns underline the importance of tailoring oral health communication strategies to generational differences.

Dietary behaviors during Ramadan also remain a critical consideration. Cultural traditions involving sweet foods during social and religious gatherings continue to influence sugar consumption patterns despite general health awareness. Previous research has shown that even moderate but frequent sugar intake can contribute to enamel demineralization and increased caries risk (23). The findings therefore reinforce the need for preventive messaging emphasizing timing, moderation, and protective oral hygiene practices during fasting periods.

### 4.2 Comparison with existing literature

Overall, the study findings are consistent with regional and international evidence examining oral health behaviors during Ramadan and within Muslim populations. Studies conducted in Saudi Arabia and Kuwait reported similar associations between education level, oral health awareness, and hygiene practices during fasting (20, 21). Generational differences identified in the present study also align with previous observations that younger individuals demonstrate lower adherence to preventive oral care during fasting (4).

International comparisons further reveal contextual differences in determinants of oral health awareness. Research from the United Kingdom suggests that structured public health campaigns can strongly influence oral hygiene behavior independent of educational level (13). In contrast, the present findings indicate that targeted Ramadan-specific campaigns remain relatively limited, despite a generally well-educated population. Similar observations have been reported globally, emphasizing that continuous reinforcement through organized health communication channels remains essential even within knowledgeable populations (14).

Cultural influences on dietary habits observed in this study mirror trends reported across Middle Eastern societies, where festive traditions are frequently associated with increased consumption of sugary foods19. Rather than representing barriers alone, such traditions may provide valuable opportunities for culturally embedded oral health promotion. Previous research has suggested that aligning preventive health messages with community and religious events enhances public engagement and behavioral acceptance (3).

Attitudinal patterns identified among participants also correspond with behavioral typologies described in earlier studies (22), where individuals may be categorized as proactive health maintainers or cautious decision-makers influenced by spiritual concerns. The coexistence of these attitudes reflects societies undergoing rapid social and cultural transition while maintaining strong religious identity.

### 4.3 Implications for oral health promotion

The findings carry important implications for oral health promotion strategies within Qatar and similar cultural settings. Persistent misconceptions regarding oral hygiene during fasting highlight the necessity of integrating religious guidance with scientific health education. Collaborative initiatives involving health authorities, dental professionals, and Islamic scholars may strengthen public confidence and clarify permissible oral care practices during Ramadan, as demonstrated in successful faith-based health campaigns implemented in neighboring Gulf countries3.

Educational interventions should extend beyond knowledge dissemination toward addressing behavioral beliefs and cultural perceptions. Integrating oral health education into mosques, community programs, and school initiatives during Ramadan may enhance accessibility of preventive messages across diverse demographic groups. Evidence suggests that culturally adapted communication strategies improve acceptance and long-term adherence to preventive behaviors (14).

Youth-focused interventions are particularly important given the influence of dietary habits established early in life. Encouraging moderation rather than restriction of traditional foods may provide a realistic and culturally acceptable approach to reducing sugar-related oral health risks. Schools, youth centers, and community platforms represent strategic environments for delivering such interventions before and during Ramadan.

Digital communication channels also present significant opportunities for outreach. The preference for official social media platforms as health information sources supports expanding verified online educational campaigns. Short, visually engaging content endorsed by trusted religious and healthcare authorities may effectively counter misconceptions while reaching broad population segments.

Interdisciplinary collaboration remains essential for sustainable oral health promotion during fasting periods. Partnerships between dental professionals, nutritionists, public health specialists, and religious leaders can facilitate comprehensive community programs addressing behavioral, dietary, and spiritual dimensions of health practice (22).

### 4.4 Integration within the broader public health context

The present findings contribute to a broader understanding of health behavior within fasting societies and support the applicability of knowledge, attitude, behavior (KAB) frameworks in culturally influenced environments. Consistent with global public health models, informational interventions alone appear insufficient unless psychological, social, and cultural determinants are simultaneously addressed1. Incorporating cultural context into health promotion aligns with international recommendations advocating culturally responsive preventive strategies.

By leveraging community traditions, educational institutions, and digital communication platforms, health authorities may foster sustained oral health behaviors extending beyond Ramadan itself. Such culturally grounded approaches have the potential to improve preventive care engagement, reduce oral disease burden, and strengthen integration between religious practice and public health promotion.

Collectively, the findings suggest that oral health behavior during Ramadan cannot be explained solely through knowledge acquisition but must be understood as a culturally embedded behavioral system shaped by religious interpretation, social norms, and collective dietary practices.

### 4.5 Study strengths and novel contributions

#### 4.5.1 Key findings and study highlights

The present study provides several key insights into oral health behavior during Ramadan fasting:

- Knowledge alone was not sufficient to predict oral hygiene behavior during Ramadan.
- Positive attitudes toward oral health acted as a critical mediator influencing brushing practices.
- Religious misconceptions regarding dental care during fasting persisted even among educated participants.
- Sugar consumption patterns appeared primarily culturally driven rather than knowledge dependent.

The study extends the Knowledge, Attitude, and Behavior framework by demonstrating that cultural and religious context functions as an external behavioral modifier influencing the translation of knowledge into practice.

#### 4.5.2 Study strengths and novel contributions

This study introduces several novel contributions that advance understanding of oral health behavior within fasting populations.

First, the research presents one of the first comprehensive Ramadan oral health behavioral models developed in Qatar, positioning fasting as a culturally specific determinant of preventive health behavior.

Second, the study integrates the Knowledge, Attitude, and Behavior (KAB) framework with dietary behavior analysis, offering a multidimensional approach that simultaneously examines oral hygiene practices and sugar consumption patterns during Ramadan.

Third, the findings identify attitude as a central behavioral mediator linking knowledge with oral hygiene practices, highlighting the importance of belief systems and religious interpretation in shaping preventive health actions.

Fourth, culturally embedded social traditions, including Garangao celebrations, are recognized as contextual factors associated with oral health risk behaviors, demonstrating how cultural events may influence dietary exposure while also providing opportunities for culturally tailored health promotion.

Finally, the study provides faith-aligned public health implications by demonstrating that oral health promotion can be effectively harmonized with religious observance, supporting culturally sensitive and religiously endorsed preventive strategies applicable to Muslim communities locally and globally.

### 4.6 Limitations

Although this study provides comprehensive insights, several limitations should be acknowledged. First, the cross-sectional design captures behaviors and attitudes at a single point in time, limiting causal interpretation of the findings. Longitudinal investigations are needed to better understand behavioral changes before, during, and after Ramadan.

Second, the relatively modest sample size (*n* = 199) may limit statistical generalizability to the wider population. While the sample was sufficient for exploratory behavioral analysis, larger multi-center studies are recommended to confirm the observed associations across broader demographic groups.

Third, participants were recruited using a convenience online sampling approach, which may introduce selection bias by favoring individuals who are digitally active or more health conscious. Nevertheless, online recruitment enabled access to fasting participants during a time-sensitive religious period, allowing examination of Ramadan-specific behaviors that are otherwise difficult to capture.

Fourth, gender distribution was not collected as a demographic variable in the survey instrument; therefore, gender-based comparisons could not be performed. This may limit interpretation of potential sex-related differences in oral health behaviors during Ramadan.

Fifth, the study relied primarily on self-reported quantitative data, which may introduce recall or social desirability bias. Anonymous participation and standardized survey procedures were applied to minimize reporting bias and enhance data reliability.

Finally, clinical oral examinations were not included; therefore, behavioral findings could not be directly linked to clinical oral health outcomes. Future research integrating behavioral assessment with clinical indicators would strengthen evidence regarding oral health outcomes during fasting.

### 4.7 Recommendations for future research

Future research should build upon the findings of this study by adopting mixed method approaches that combine quantitative surveys with qualitative methods such as focus groups to explore personal experiences and religious interpretations influencing oral health behavior. Expanding sample diversity to include youth, older adults, and non-Qatari residents may enhance generalizability across population groups. In addition, longitudinal designs are needed to examine whether improvements in oral health knowledge and attitudes translate into sustained behavioral change beyond the Ramadan period.

Overall, the study demonstrates that effective oral health promotion during Ramadan requires behavioral, cultural, and faith-integrated strategies rather than knowledge-based education alone.

## 5 Conclusion

This study sought to provide an in-depth investigation of oral health knowledge, behaviors, attitudes, and sugar consumption among individuals living in Qatar during the Ramadan period. It highlighted the intricate interactions between cultural, religious, and behavioral elements of oral hygiene. The obtained findings disclosed that although many participants uphold consistent oral hygiene practices and exhibited a strong awareness of the importance of dental care, existing misconceptions and cultural traditions persisted to influence behaviors. It was noted that education emerged as the leading significant predictor of oral health knowledge since higher education levels had a correlation with better understanding of acceptable dental treatments during fasting hours. These notable gaps emphasized the continued influence of religious and cultural interpretations on health behavior, even within well-educated individuals. Additionally, the analysis of attitudes led to the exposure of two distinct clusters, namely Proactive and Cautious, representing those who embrace preventive oral care when fasting and those who evade it because of the perceived religious restrictions. This indicates that health interventions should seek to not only educate but also aim at reshaping perceptions for alignment with faith and health goals.

This current study establishes that Qatar's fasting population exhibits admirable oral hygiene awareness and adherence, but there persist some challenges persist. Education and positive attitudes emerged as fundamental in upholding sustainable, healthy behaviors, although sugar consumption persists to be a culturally entrenched challenge. The findings advocate for faith-aligned and community-driven health promotion strategies with an aim to clarify religious rulings, endorse preventive dental care, and promote balanced dietary practices. Therefore, it is in uniting medical, educational, and religious efforts that policymakers can nurture a holistic approach to oral health culture that is in alignment with spiritual devotion and physical well-being to ultimately enhance public health outcomes during and beyond Ramadan.

### 5.1 Policy and practical implications

From a public health perspective, the obtained findings have notable implications for developing culturally sensitive oral health promotion programs within Qatar and other Muslim communities. Health authorities are expected to work closely with religious scholars and community leaders since the collaborative efforts lead to the development of educational materials that clarify permitted oral care practices during fasting. Also, the Ministry of Public Health and dental associations must prioritize faith-based campaigns emphasizing the essence of preventive care, encouraging regular dental visits, and dispelling misconceptions regarding toothpaste, mouthwash, and dental procedures. Lastly, provided that over half of the participants perceived a lack of sufficient awareness from official sources, policymakers need to invest in widespread communication strategies that can exploit the potential of digital platforms.

### 5.2 Recommendations for future research

Future research must utilize the obtained findings of this study as a foundation to advance exploration by employing mixed-method approaches, which can incorporate surveys with focus groups to discuss and explore personal experiences and religious interpretations that have influenced oral health behavior. Expanding the sample to consider a more diverse population, especially youth, elderly, and non-Qatari residents, can enhance the generalizability of derived results. Moreover, longitudinal studies can assess whether oral health knowledge and attitudes result in sustained behavioral improvements after Ramadan.

## Data Availability

The raw data supporting the conclusions of this article will be made available by the authors, without undue reservation.
